# Supplementary material for: Giant thermal expansion of a two-dimensional supramolecular network triggered by alkyl chain motion
Source: Commun Mater. 2020 Feb 17;1(1):8. doi: 10.1038/s43246-020-0009-2 (PMC7099928; doi:10.1038/s43246-020-0009-2)
Supplement: Supplementary file 1 — Supplementary Information [file 43246_2020_9_MOESM1_ESM.pdf]

Supplementary Material for: Giant thermal expansion of a 2D supramolecular network triggered by interdigitation

Sebastian Scherb,[†,§] Antoine Hinaut,[*,†,§] Rémy Pawlak,[†] J.G. Vilhena,[*,†] Yi Liu,[‡] Sara Freund,[†] Zhao Liu,[†] Xinliang Feng,[¶] Klaus Müllen,[‡] Thilo Glatzel,[†] Akimitsu Narita,[‡] and Ernst Meyer[*,†]

†*Department of Physics, University of Basel, Klingelbergstrasse 82, 4056, Basel, Switzerland*

‡*Max Plank Institute for Polymer Research, Ackermannweg 10, 55128, Mainz, Germany*

¶*Faculty of Chemistry and Food Chemistry, TU Dresden, Mommsenstrasse 4, 01069, Dresden, Germany*

§*These authors contributed equally to this work.*

* E-mail: antoine.hinaut@unibas.ch; guilhermevilhena@gmail.com; ernst.meyer@unibas.ch

Supplementary Figure 1: Large scale STM of isolated spoked wheel

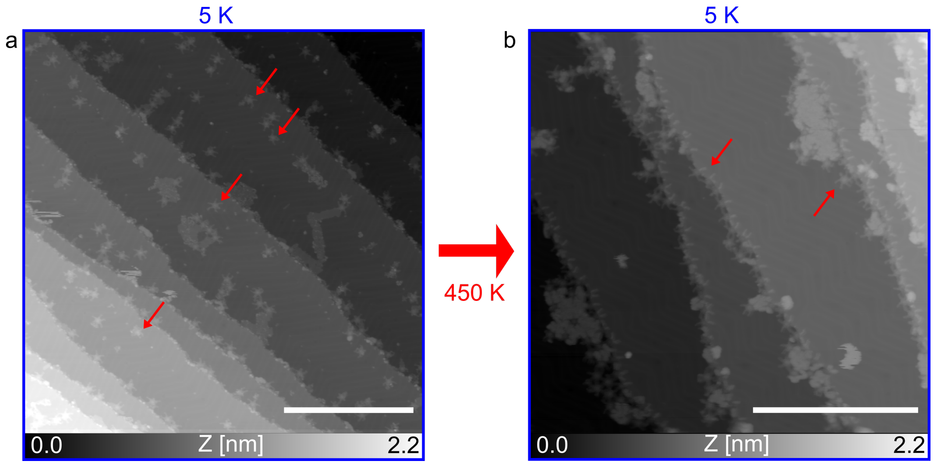

Supplementary Figure 1: **Isolated spoked wheel molecules on Au(111).** (a) Topography STM image at 5 K of SW molecules on Au(111) surface after spray deposition onto a surface maintained at room temperature, prior to annealing. (b) Topography STM image of similar density area after annealing at 450 K, observed after microscale tip displacement from Fig. 2 a. Isolated SW molecule on Au(111) surface and step edges at 5 K are visible as pointed with red arrow. Scale bar is 50 nm.

Supplementary Figure 2: Large scale assemblies on Au(111)

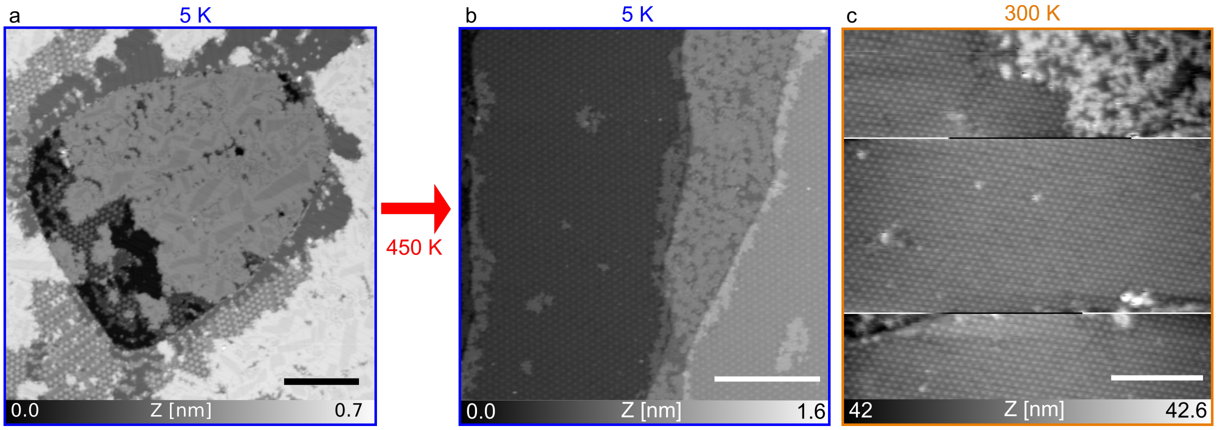

Supplementary Figure 2: **Large scale assemblies on Au(111).** (a) Topography STM image of SW molecule high density area on Au(111) surface at 5 K after spray deposition and prior to annealing. (b) Topography STM image of SW molecule assemblies on Au(111) surface at 5 K after annealing at 450 K. (c) Topography AFM image at 300 K of SW molecule assemblies on Au(111) surface after annealing at 450 K. Scale bars are 50 nm.

Supplementary Note 1: Molecular Dynamics simulation protocol

The simulation protocol is a two stage process. The first stage concerning single spoked wheel (SW) over Au(111) and the second concerning SW trimer over Au(111).

Single spoked wheel molecule

The starting configuration of the SW molecule was built using Avogadro software. The structure was then relaxed via an energy minimization simulation using as a stop criteria that the root-mean-square of the cartesian elements of the energy gradient has to be smaller than 1.0×10^{-4} kcal/mole-Å; a truncation of the non-bonded interactions consistent with all the other simulations, see in Methods the molecular dynamics (MD) simulation details, and a hybrid steepest decent (100 steps) and conjugated gradient (500 steps) procedure as implemented in AMBER18.[1] Prior to the energy minimization, atom-types, charges, Lennard-Jones parameters, bonds, angles and dihedral assignment of the SW molecule is assigned accordingly to GAFF[2] force-field with the use of ANTECHAMBER software suite.[3]

The final configuration of the molecule was placed 1 nm away from the Au(111) surface as shown in Supplementary Figure 3a,b. Then, the molecule was allowed to adsorb to the surface in a 10 ns long MD simulation performed at 300 K. The final adsorption configuration is shown in Supplementary Figure 3c,d. Starting from this configuration, 1 μs long MD simulations at four different temperatures were performed.

Spoked wheel trimer assembly

The thermal stability of the SW molecular assembly was evaluated by using three SW, packed in a conformation that matches the inter-molecular distance measured in the low temperature experiments of small molecular assemblies. Such a molecule trimer was imaged by LT STM,

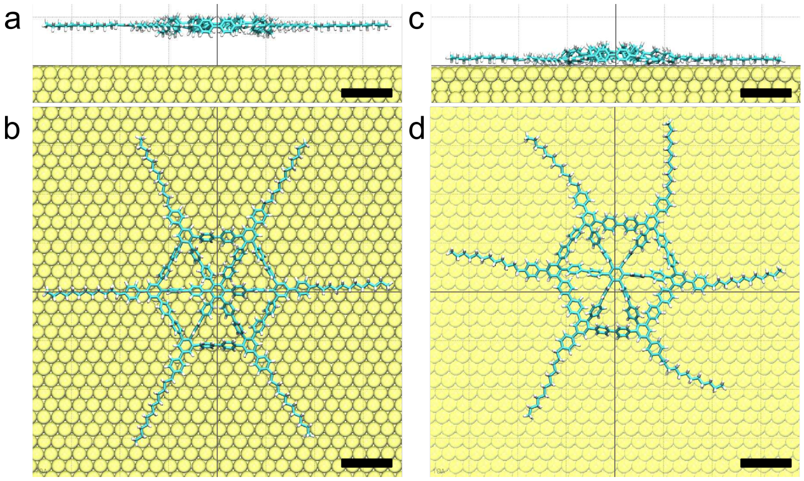

Supplementary Figure 3: **Starting configuration of a single SW over Au(111).** Side (a) and top (b) view prior to adsorption. Side (c) and top (d) views after adsorption to Au(111) over a 10 ns long NVT simulation at 300 K. The Au(111) atoms are represented in yellow and the atoms of the molecule are represented in cyan for the carbon and white for the hydrogen. Scale bar is 1 nm long.

as visible in Supplementary Figure 4a, and gives an average distance between molecules of 3.8 to 4.2 nm (which is slightly larger than the average distance in large molecular assemblies shown in Fig. 2 – d=3.5 nm).

This small system was used, since for an all atom simulation point of view, it is impossible to perfectly reproduce the large molecular assemblies observed experimentally. This is because of the large system that has to be considered and mostly since the time scales accessible in the simulations (i.e. μs) are several orders of magnitude smaller than the ones existing in the experiments (i.e. minutes). Additionally, larger assemblies will certainly take longer to equilibrate as a result of a larger phase space and the required concerted motion of the assembly as a whole, which at the experiments time scale (minutes) would not play an important role. Still much can be learned about the assembly dynamics with a careful choice of the simulation parameters as outlined and justified in the following.

Firstly, we had to build an assembly that matches the compact structure of small molecular assemblies - which best describes our simulation setup - such as the one shown in Supplementary Figure 4a. The reason for this is as follows. As the system is cooled down

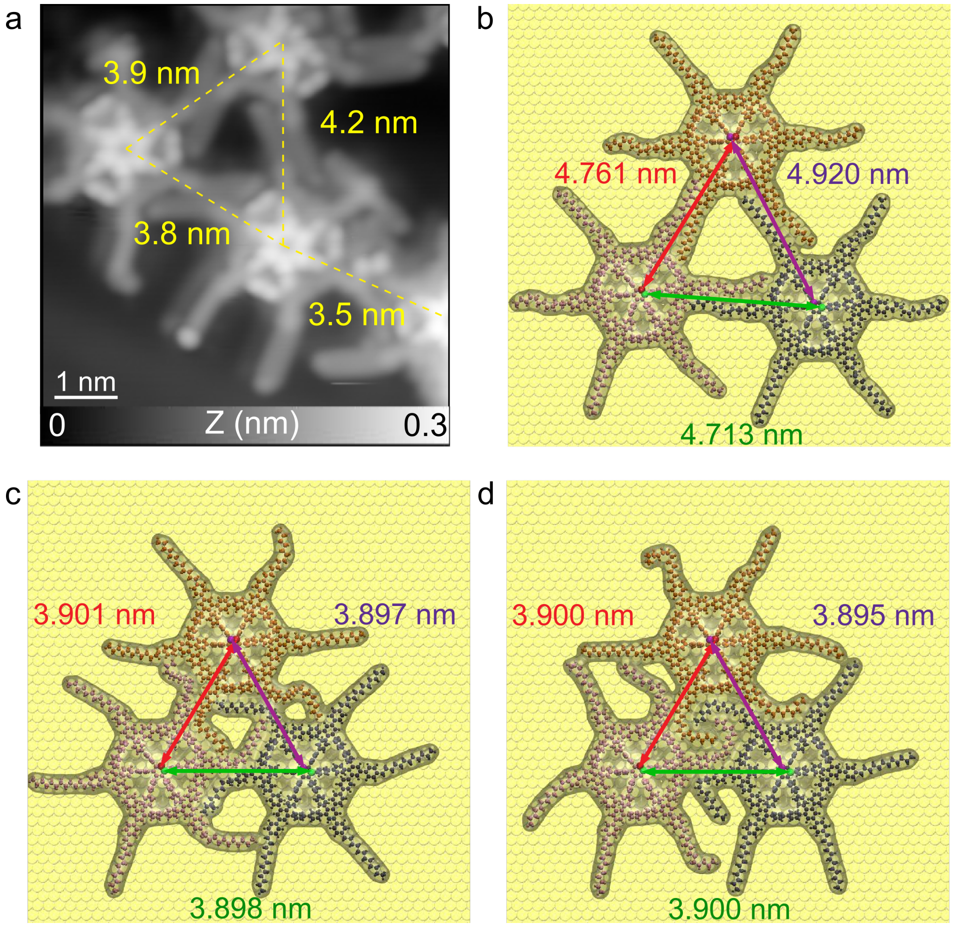

Supplementary Figure 4: **Building a SW trimer assembly based on the experimental findings.** a) Topography STM image at 5 K of a SW trimer formed on Au(111) surface. b) Starting configuration built from equilibrated single SW MD simulations at 300 K; c) Configuration obtained after 1 ns of MD simulations at 300 K with an harmonic restrain in the centre of the molecules imposing a distance of 3.9 nm between the cores. d) Configuration obtained after a 10 ns long MD simulation at 450 K, while keeping the distances between the molecules fixed, in order to equilibrate the conformation of the arms located in-between the molecules.

the thermal energy available for the molecules to diffuse is decreased. Consequently, the slip events that would give rise to the compaction of the network become less and less frequent, which otherwise said means that they need much more time to occur. This ultimately renders the simulation of compaction unfeasible as the time needed to reach a thermal equilibrium is much longer than the accessible μs long simulations tractable at the MD level. Note that as the molecules diffuse inwards in order to give rise to a more compact structure they also form an energetically more favourable configuration as shown in Supplementary Figure 4d.

Interestingly, an indirect evidence for this effect can be found in our experiments as small molecular assemblies (see Supplementary Figure 4a) have a bigger core-core distance with respect to the large molecular assemblies (see Figure 2).

For this reason, a compact structure mimicking a small molecular assembly (shown in Supplementary Figure 4a) was chosen as starting point and then heated up. The heating process, gives rise to an increase in thermal fluctuations and kinetic energy reducing the simulation time required to reach a thermal equilibrium. Still, it must be large enough so the diffusion events are frequent enough, but not too high to promote spontaneous desorption of the molecules from the surface. For this reason, only two limiting temperatures, i.e. 5 and 450 K, were picked. At 450 K not only the molecules do not desorb (confirmed experimentally) but also have a small body mobility, as shown in the single molecule simulations (see Fig.3 of the main manuscript). Such small diffusion at high temperatures is most likely related with the large interaction area of the molecule core with the Au(111) substrate. The low diffusion of the core at 450 K, in the assembly allows the differentiation of spontaneous body diffusion with the one resulting from the "pushing" of the neighbouring chains.

In order to build a low temperature SW trimer assembly, a configuration sampled from the equilibrium trajectory of the production run from stage 1 at 300 K, (extracted at 700 ns) was used. From this starting configuration, an assembly of three SW molecules is built, as shown in Supplementary Figure 4b. In this assembly the distance between the cores is much larger than the one reported in the low temperature experiments, in Supplementary Figure 4a, a choice taken in order to avoid any spurious steric repulsion effect between the chains of the different molecules. Then, a 1 ns long MD simulation at 300 K imposing a distance harmonic restrain between the cores of the three different molecules was performed. The restrain consists of three springs with a stiffness of 50 kcal/molÅ^2 which impose a distance of 3.9 nm between equivalent core atoms of neighbouring molecules. The resulting configuration is shown in Supplementary Figure 4c. Finally, the system was annealed at 420 K during

10 ns, whilst maintaining the distance between the cores via the aforementioned restrain. The final configuration is shown in Supplementary Figure 4d, and it corresponds to the starting configuration of the simulations performed at 5 and 450 K shown in the Figure 4 of the main text. The details of these production runs are provided in the Methods section of the main text.

Supplementary Note 2: Stereochemically induced spoked wheel threefold symmetry

In Figure 3a of the main text, the final configurations obtained for a single SW deposited over an Au(111) surface at four different temperatures is shown. The topographic image of each of these configurations shows that, regardless the temperature considered, the molecular body exhibits a three fold symmetry. Such symmetry is apparent in the most elevated region of the molecule and is also observed experimentally using STM and constant-height AFM (see Figure 1 of the main manuscript).

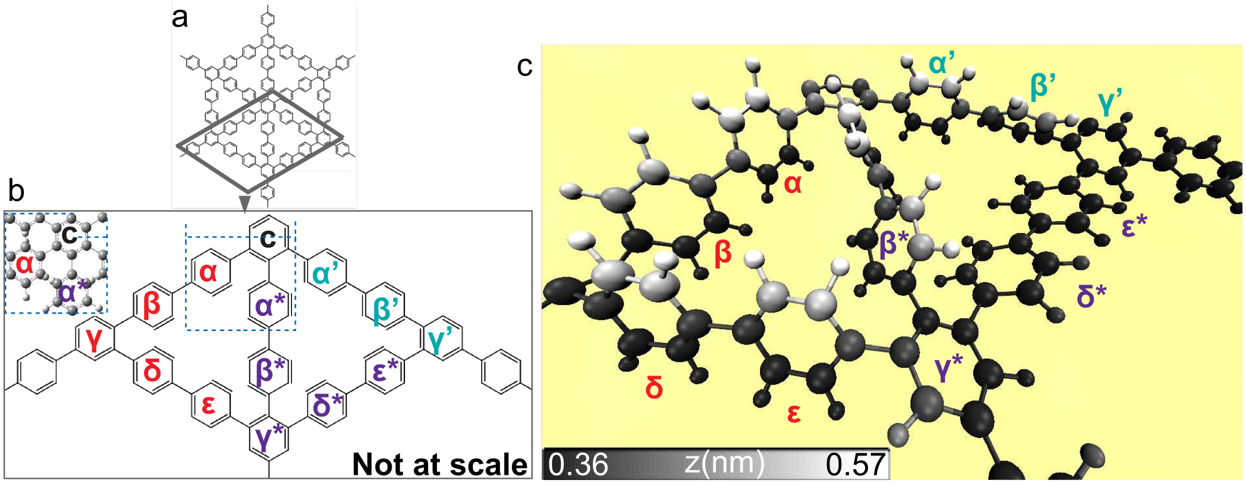

Supplementary Figure 5: **Three fold symmetry of the SW adsorbed on Au(111) at different temperatures.** (a) Schematic representation of the SW were the six-fold symmetry is visible. (b) Zoom on the molecule scheme. Each of the aromatic rings composing the central region of the molecule is named, for a better explanation of the steric effects. (c) Perspective view of the zoom in b. Tilting of the aromatic rings is visible to show the three fold symmetry and the steric hindrance.

The threefold symmetry of the molecule can be understood in terms of steric hindrance between the different parts. The ad-hoc nomenclature introduced in Supplementary Figure 5b to name each of the aromatic rings is used to explain the symmetry and better explain the origin of the different steric repulsions. Given the three fold symmetry, the N spoke is identical to the N+2 (shown in Supplementary Figure 5b as cyan aromatic rings). First, the central region of the molecule cannot lay flat on the surface. This results from steric repulsion between the α and α^* attached to the core (c) aromatic ring. This effect is shown in the inset of Supplementary Figure 5b, where with planar conformation the hydrogen of α almost ends up on top of the neighbouring carbon atom of α^*. Major steric repulsion would prevent such conformation. As a result, all aromatic rings attached to the core, i.e. α and α^*, are in an upright position. Consequently, this also results in an upright conformation of the β and β^* rings. In this case the carbon connecting to the α is always higher than the carbon connecting to the γ ring (see e.g. the β^* in Supplementary Figure 5c). Then, depending on the tilting direction of the β ring the side chain composed by δ and ϵ may lay flat or not on the surface. A flat conformation increases the interaction with the surface, but this comes at the cost of a steric repulsion between the hydrogen atoms of β with hydrogen atoms of δ and ϵ. This may be better understood through the example shown in Supplementary Figure 5c, where δ^* hydrogen forces β^* to adopt a tilted conformation that now renders the planar adsorption of ϵ sterically inhibited. For this reason ϵ is adsorbed in an upright position. In order to reduce twist between neighbouring molecules, it is preferable that both δ and ϵ adsorb equivalently, i.e. either upright or flat lying. The alternation of this upright and flat-on adsorption of the side chains driven by the steric repulsion between the spokes gives rise to the three fold symmetry of the molecule.

Supplementary Note 3: Thermal expansion of spoked wheel

Thermal expansion for single spoked wheel

In order to characterize the mobility of the different molecule part, we used the mean square displacement of the body and of the chain parts separately. The body is represented by the centre of mass of the central aromatic ring, Supplementary Figure 6a as a purple bead. The chain is represented by its last carbon atom, Supplementary Figure 6a as orange bead, and all chains are considered identical. From the Supplementary Movies 3-5, the body mobility is about the same regardless the temperature considered. The mean square displacement (MSD) of the body shown in the top panel of Supplementary Figure 6a shows some differences between different temperatures. These are related to a single displacement event occurring at the beginning of the simulation related to a motion of the molecule which improves its registry with the substrate. For this reason, the MSD increases from 0 to almost 2 Å initially for some values of temperature. In these cases, the molecules remained anchored to the position then adopted for a period larger than 800 ns giving incorrect MSD values. Instead, by looking at the standard deviation of the MSD (bottom panel of Supplementary Figure 6a), it is observed to remain almost zero for all temperatures, which is more consistent with what was observed in the movies aforementioned. This standard deviation of the MSD, i.e. δMSD, provides a better picture of the fluctuations occurring during the simulations and was chosen as main parameter.

When comparing the fluctuations of the chains and the body, hereafter referring to δMSD, it can be seen that the chains have a much higher mobility than the bodies. More importantly, whereas the body fluctuations seem to remain virtually unaltered with increasing temperature, this is certainly not the case for the arms, whose fluctuations increase with increasing temperature as shown in Supplementary Figure 6b.

In order to provide a geometric picture to the chain fluctuations, the angle variation of

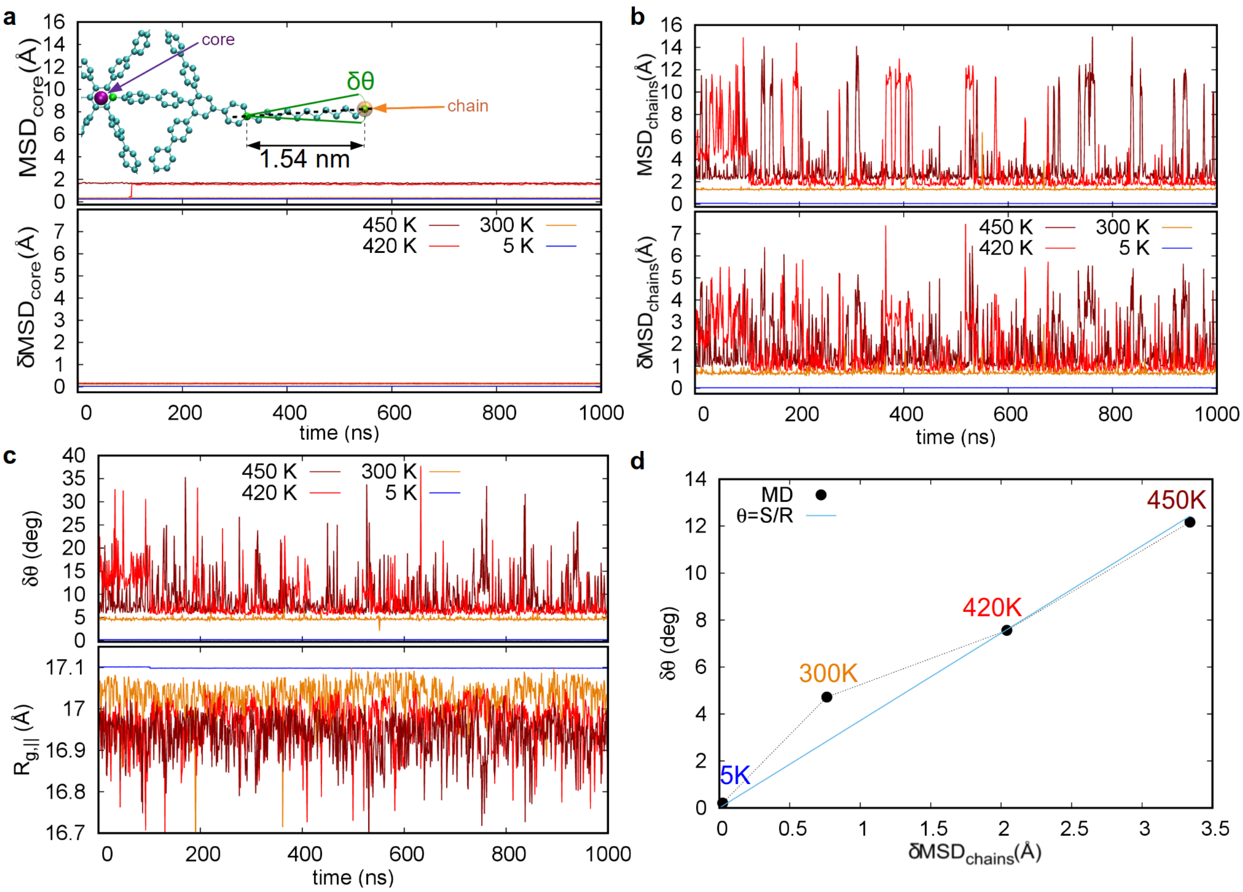

Supplementary Figure 6: **Fluctuations of the chains and body of a single SW molecule on Au(111) as predicted from MD simulations.** (a) Time averaged Mean square displacement (MSD) over a time frame of 1 ns its standard deviation as a function of time. (b) Same as (a) evaluated for the arms fluctuation. (c) Time averaged (over 1 ns) angle fluctuations and parallel gyration tensor component as a function of time. (d) Correlation between the angle and mean-square-displacement fluctuations computed in the time frame corresponding to the last 100 ns of simulation. The inset in (a) shows the atoms over which the MSD and its standard deviations are computed, i.e. the body and the chains.

the chain, represented in the inset of Supplementary Figure 6a as $\delta\theta$, is analysed. As it happened with the δMSD, the angle fluctuations also increase with increasing temperature thus showing how higher temperatures promote a higher bending of the chains. This means that the effective radii of the molecule also decrease with increasing temperature, as the larger the chain bending the smaller the effective radii of the molecule should be. This is nicely described by the sum of the diagonal terms of the radius of gyration tensor parallel to the surface, i.e. x and y, which is represented in the bottom panel Supplementary Figure 6c.

This ultimately shows that at single molecule level, the SW has a negative thermal expansion, as it becomes smaller with increasing temperature.

At last, it is also instructive to look at the correlation between the angle and the displacement fluctuations of the chain. One can think of it as δMSD being the arc, and $\delta\theta$ the associated angle. In fact plotting the former as a function of the latter an almost linear relation is found, see Supplementary Figure 6d, where the slope is defined by the length of the arm. Thus, the arm displacements define an arc S, which is directly proportional to the angle change θ and the length of the arm, i.e. $S=R\theta$. This relation shows that most of the fluctuations stems from a bending of the chain and not a major structural modifications of the dihedrals defining the alkyl chain that could also give rise to major displacements.

Thermal expansion for a molecular assembly

In case of molecular assemblies, the increase of the chain mobility with the temperature does not result in the negative thermal expansion as for single molecule (Supplementary Figure 6c). The opposite behaviour, i.e giant thermal expansion, is observed as shown in Figure 2 of the article and discussed in the corresponding part, "Influence of interdigitated dodecyl chains on thermoresponse".

Molecular Assembly stability at high temperatures (>500 K)

Contrary to covalently bonded materials, molecular assemblies are held together by much weaker interactions, and are therefore prone to a swift degradation at relatively lower temperatures. In fact, in our experiments we observed that after annealing at $T > 500$ K the molecules either desorbed from the surface or decomposed giving rise to small fragments on the surface. On the basis of this result, and given that our interest lays on understanding the thermoresponse of a molecular assembly we found unnecessary to complement these findings with additional simulations.

Supplementary Note 4: Thermal expansion of hexabenzocoronene molecular assemblies

In a precedent study,[4] the compatibility of the molecular spray deposition method with cleanliness conditions necessary for high resolution SPM in UHV was shown. There, the HBC-6$C_{12}H_{25}$ molecule, Figure 7a, adsorption on the Au(111) surface was studied on same RT and LT systems than in the present study. Similar to SW molecules, the formation of large island was found and imaged both at RT and LT. Topography STM image acquired at 5 K on a large island of HBC-6$C_{12}H_{25}$ is visible in Figure 7b. Topography nc-AFM image of a similar island at RT is visible in Figure 7c.

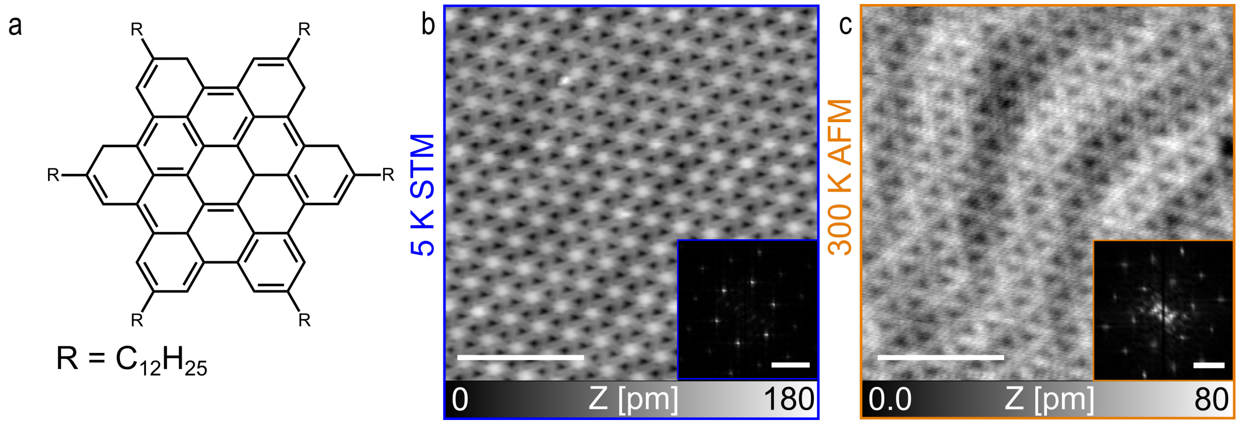

Supplementary Figure 7: **HBC-6$C_{12}H_{25}$ molecule on Au(111).** a) Molecular scheme. b) STM topography image of a large island of HBC-6$C_{12}H_{25}$ formed on Au(111). c) nc-AFM topography of a similar HBC-6$C_{12}H_{25}$ island. d) and e) FFT images of b) and c) respectively. Scale bars: topographies: 10 nm; FFT: 0.5 $nm^{-}1$

Extracted from topography images and their corresponding FFT, the lattice values are $a_{5\ K} = 2.2$ nm and $a_{300\ K} = 2.6$ nm.[4] The corresponding thermal coefficient is $\alpha = 520 \times 10^{-6}\mathrm{K}^{-1}$.

HBC pristine molecule on Au(111) surface was also investigated in previous studies.[5,6] Values presented in the table 1 of the main manuscript are extracted from these two references.

References

(1) Case, D. et al. AMBER 2018, University of California, San Francisco. (2018)

(2) Wang, J., Wolf, R. M., Caldwell, J. W., Kollman, P. A., Case, D. A. Development and Testing of a General Amber Force Field. *J. Comput. Chem. 25*, 1157–1174 (2004).

(3) Wang, J., Wang, W., Kollman, P. A. & Case, D. A. Automatic atom type and bond type perception in molecular mechanical calculations. *J. Mol. Graph. Model. 25*, 247–260 (2006).

(4) Hinaut, A. et al. Electrospray deposition of structurally complex molecules revealed by atomic force microscopy. *Nanoscale 10*, 1337–1344 (2018).

(5) Meissner, M. et al. Flexible 2D Crystals of Polycyclic Aromatics Stabilized by Static Distortion Waves. *ACS Nano 10*, 6474–6483 (2016).

(6) Wagner, C. et al. Repulsion between molecules on a metal: Monolayers and submonolayers of hexa-peri-hexabenzocoronene on Au(111). *Phys. Rev. B 81*, 035423 (2010).
