# Supplementary material for: Giant thermal expansion of a two-dimensional supramolecular network triggered by alkyl chain motion
Source: Commun Mater. 2020 Feb 17;1(1):8. doi: 10.1038/s43246-020-0009-2 (PMC7099928; doi:10.1038/s43246-020-0009-2)
Supplement: Supplementary file 3 — Description of Additional Supplementary Files [file 43246_2020_9_MOESM3_ESM.pdf]

Supplementary Movies Information for: Giant thermal expansion of a 2D supramolecular network triggered by interdigitation

Sebastian Scherb,[†,§] Antoine Hinaut,[*,†,§] Rémy Pawlak,[†] J.G. Vilhena,[*,†] Yi Liu,[‡] Sara Freund,[†] Zhao Liu,[†] Xinliang Feng,[¶] Klaus Müllen,[‡] Thilo Glatzel,[†] Akimitsu Narita,[‡] and Ernst Meyer[*,†]

†*Department of Physics, University of Basel, Klingelbergstrasse 82, 4056, Basel, Switzerland*

‡*Max Plank Institute for Polymer Research, Ackermannweg 10, 55128, Mainz, Germany*

¶*Faculty of Chemistry and Food Chemistry, TU Dresden, Mommsenstrasse 4, 01069, Dresden, Germany*

§*These authors contributed equally to this work.*

* E-mail: antoine.hinaut@unibas.ch; guilhermevilhena@gmail.com; ernst.meyer@unibas.ch

Supplementary Movies

Supplementary Movie 1: Molecular Dynamics simulation of three spoked wheel molecules on Au(111) at 5 K for 1 μs.

Supplementary Movie 2: Molecular Dynamics simulation of three spoked wheel molecules on Au(111) at 450 K for 1 μs.

Supplementary Movie 3: Molecular Dynamics simulation of a single spoked wheel molecule on Au(111) at 5 K for 1 μs.

Supplementary Movie 4: Molecular Dynamics simulation of a single spoked wheel molecule on Au(111) at 300 K for 1 μs.

Supplementary Movie 5: Molecular Dynamics simulation of a single spoked wheel molecule on Au(111) at 450 K for 1 μs.
